# Targeting cellular senescence based on interorganelle communication, multilevel proteostasis, and metabolic control

**DOI:** 10.1111/febs.15631

**Published:** 2020-12-08

**Authors:** Maria Cavinato, Corina T. Madreiter‐Sokolowski, Sabrina Büttner, Markus Schosserer, Werner Zwerschke, Sophia Wedel, Johannes Grillari, Wolfgang F. Graier, Pidder Jansen‐Dürr

**Affiliations:** ^1^ Institute for Biomedical Aging Research Leopold‐Franzens Universität Innsbruck Austria; ^2^ Center for Molecular Biosciences Innsbruck (CMBI) Leopold‐Franzens Universität Innsbruck Austria; ^3^ Department of Health Sciences and Technology Institute of Translational Medicine Swiss Federal Institute of Technology (ETH) Zurich Switzerland; ^4^ Molecular Biology and Biochemistry Gottfried Schatz Research Center Medical University of Graz Austria; ^5^ Institute of Molecular Biosciences University of Graz Austria; ^6^ Department of Molecular Biosciences The Wenner‐Gren Institute Stockholm University Sweden; ^7^ Christian Doppler Laboratory for Skin Multimodal Analytical Imaging of Aging and Senescence Institute of Molecular Biotechnology University of Natural Resources and Life Sciences Vienna Austria; ^8^ Austrian Cluster for Tissue Regeneration Medical University of Vienna Austria; ^9^ Ludwig Boltzmann Institute for Experimental and Clinical Traumatology Vienna Austria; ^10^ BioTechMed Graz Austria

**Keywords:** calcium signaling homeostasis, caloric restriction mimetic, interorganellar connectivity, lysosome, mitochondria, mitophagy, proteostasis, RNA modification, senescence, translational control

## Abstract

Cellular senescence, a stable cell division arrest caused by severe damage and stress, is a hallmark of aging in vertebrates including humans. With progressing age, senescent cells accumulate in a variety of mammalian tissues, where they contribute to tissue aging, identifying cellular senescence as a major target to delay or prevent aging. There is an increasing demand for the discovery of new classes of small molecules that would either avoid or postpone cellular senescence by selectively eliminating senescent cells from the body (i.e., ‘senolytics’) or inactivating/switching damage‐inducing properties of senescent cells (i.e., ‘senostatics/senomorphics’), such as the senescence‐associated secretory phenotype. Whereas compounds with senolytic or senostatic activity have already been described, their efficacy and specificity has not been fully established for clinical use yet. Here, we review mechanisms of senescence that are related to mitochondria and their interorganelle communication, and the involvement of proteostasis networks and metabolic control in the senescent phenotype. These cellular functions are associated with cellular senescence in *in vitro* and *in vivo* models but have not been fully exploited for the search of new compounds to counteract senescence yet. Therefore, we explore possibilities to target these mechanisms as new opportunities to selectively eliminate and/or disable senescent cells with the aim of tissue rejuvenation. We assume that this research will provide new compounds from the chemical space which act as mimetics of caloric restriction, modulators of calcium signaling and mitochondrial physiology, or as proteostasis optimizers, bearing the potential to counteract cellular senescence, thereby allowing healthy aging.

## Graphical abstract

Cellular senescence, a stable cell division arrest caused by severe damage and stress, is a hallmark of aging in humans. Here, we review mechanisms of senescence that are related to mitochondria and their interorganelle communication, and the involvement of proteostasis networks and metabolic control in the senescent phenotype. We also explore possibilities to target these mechanisms as new opportunities for pharmacological interventions with the aim of tissue rejuvenation.

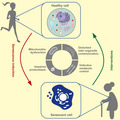

**Abbreviations**
AMPK: adenosine monophosphate‐activated protein kinase
ASC: adipose‐derived stem cells
BNIP3L/Nix: BCL2/adenovirus E1B 19 kDa protein‐ interacting protein 3‐like
CR: caloric restriction
DIRAS3: DIRAS family GTPase 3
Dnm1l: dynamin 1 like (also known as Drp1)
DR: dietary restriction
eIF4G: eukaryotic translation initiation factor 4G
ER: endoplasmic reticulum
ETC: electron transport chain
FAHD1: FAH domain containing protein 1
FUNDC1: FUN14 Domain Containing 1
IGF‐1: insulin‐like growth factor‐1
MAM: mitochondria‐associated endoplasmic reticulum membranes
MAPK: mitogen‐activated protein kinase
MCU: mitochondrial calcium uniporter
MDV: mitochondria‐derived vesicles
METTL5: methyltransferase like 5
MICU: mitochondrial calcium uptake protein
mTOR: mammalian target of rapamycin
NAD^+^
: nicotinamide adenosine dinucleotide (oxidized form)
NSUN5: NOP2/Sun RNA methyltransferase 5
PI3K: phosphoinositide 3‐kinase
PINK1: PTEN‐induced Kinase 1
PRMT1: Protein methyl transferase 1
ROS: reactive oxygen species
SASP: senescence‐associated secretory phenotype
TCA cycle: tricarboxylic acid cycle

## Introduction

**Fig. 1:**
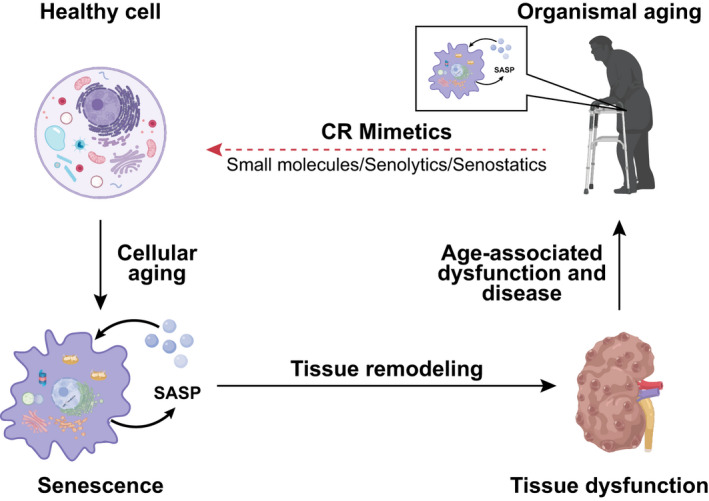
The role of senescence and its reversion in healthy aging and age‐related dysfunction and disease. With increasing age, senescent cells accumulate in several human tissues, due to a process known as cellular aging. Senescent cells secrete a plethora of extracellular proteins, lipids, and other bioactive material, collectively referred to as the senescence‐associated secretory phenotype (SASP). SASP components trigger changes in adjacent cells resulting in tissue remodeling and subsequently age‐associated tissue dysfunction of human organs, such as the kidney. Age‐associated dysfunction in several tissues contributes to organismal aging. Currently, small molecules with the potential to trigger the elimination of senescent cells (referred to as senolytics) or to dampen their detrimental influence on the organism (referred to as senostatics) are under development. Such compounds, with a potential for tissue rejuvenation, may provide new therapeutic opportunities for age‐associated dysfunctions and diseases. Based on the beneficial effects of caloric restriction (CR) on human healthspan, compounds which mimic beneficial effects of caloric restriction may be particularly suitable candidates for the development of senolytics and/or senostatics.

Aging is a complex process driving progressive decline of functionality and regenerative potential of tissues. One hallmark of aging is cellular senescence, a state of stable cell division arrest caused by severe damage and stress, leading to cellular dysfunctions among others in metabolic signaling, intra‐organelle signaling, proteostasis, and mitochondria. Senescence is involved in tissue homeostasis, embryonic development as well as inhibition of tumor progression [1]. During aging, senescent cells accumulate in multiple organs and compromise tissue function, essentially caused by the unique property of senescent cells to secrete a bunch of pro‐inflammatory and damage‐inducing molecules, commonly referred to as the senescence‐associated secretory phenotype (SASP). SASP components are causally involved in molecular and cellular changes giving rise to pathological manifestations and frailty. Hence, senescence is most likely a defining feature of human age‐related diseases, including obesity, diabetes mellitus type 2, cardiovascular disease, skin aging, and cancer [2, 3] (Fig. 1).

Although considerable progress has been achieved in the field of cellular senescence, it is still not clear whether the appearance of senescent cells is causative to or a mere consequence of the aging process. Elimination of senescent cells has been shown to extend both lifespan and health span of different organisms [2]. Still, there is an increasing demand for the discovery of new classes of small molecules, which would either avoid or postpone cellular senescence by selectively eliminating senescent cells from the body (i.e., ‘senolytics’) or by inactivating/modulating damage‐inducing properties of senescent cells (i.e., ‘senostatics/senomorphics’) [4, 5], such as the SASP.

Recent literature suggests that age‐related dysfunctions can be counteracted by senolysis in animal models; accordingly, there are attempts to eliminate senescent cells in order to delay aging. While there is evidence in favor of this hypothesis, alternative scenarios can be envisaged as well. An example might be the activation of the p16/Rb and/or p21/p53 stress response pathways, which boost key conserved molecular mechanisms driving cellular senescence. Still, one cannot exclude that in some instances, the activation of the p16/Rb and p21/p53 pathways primarily serves as marker of dysfunctional cells ready to be eliminated.

Blocking the mechanisms that cause senescence may not resolve the dysfunction but just one of its consequences. Thus, elimination of senescent cells can cause detrimental rather than beneficial effects to the organism [6, 7], which is not too surprising given the participation of cellular senescence in mammalian development [8] and during wound healing [9], a process driven by SASP components.

Several molecular and signaling pathways related to cellular dysfunction (cell cycle arrest, DNA and protein damage response, etc.) that contribute to induction and maintenance of the senescent state are conserved in evolution. Thus, simple and easy‐to‐manipulate model organisms such as the yeast *Saccharomyces cerevisiae* (*S. cerevisiae*) or the nematode *Caenorhabditis elegans* (*C. elegans*) are frequently used to elucidate fundamental aspects of cell damage and disruption of homeostasis, which promote senescence in vertebrates [10, 11, 12, 13, 14, 15]. However, there is little evidence for senescence in these simple models, which are evolutionarily quite distant from humans. Consequently, mammalian aging models, including mice, and human organoids, such as human skin equivalents and adipose spheroids, have to be used to gain insights into the translatability from simple to more complex organisms [16].

In order to overcome negative effects of cellular senescence on human health [17], it is essential to identify aging‐related pathways and mediators to elaborate senescence‐associated mechanisms, including maintenance of mitochondrial functions, interorganelle communication, proteostasis, and metabolic signaling in selected cellular and organismal aging models. The identification of pathways and cellular targets along with lead compounds may pave the way for the development of treatment strategies to counteract age‐associated cellular dysfunction and, ultimately, pathophysiological processes, to promote healthy aging [18].

## Mitochondria‐related mechanisms of senescence

Aside from being the cell's power plants, mitochondria serve as metabolic hubs, contributors to signal transduction, autophagy, and programmed cell death, and are in a unique position to mediate or modify aging‐associated processes, including cellular senescence. In fact, it has been shown that impairment of mitochondrial function is sufficient to induce senescence in a variety of cell types [19, 20, 21]. On the other hand, cells lacking mitochondria do not respond to most senescence‐inducing stress factors [22], highlighting the key role for mitochondria in senescence induction and execution.

**Fig. 2:**
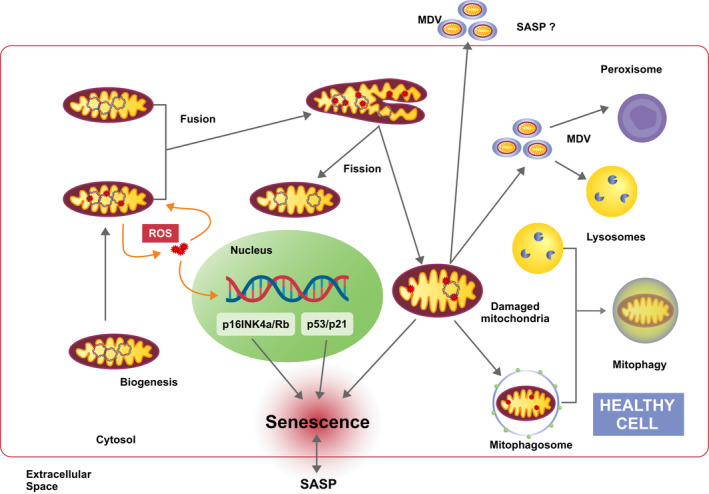
Mitochondrial transitions and interactions in cellular senescence. Mitochondria are dynamic organelles which act as the primary energy‐generating system in most eukaryotic cells. In addition, mitochondria are involved in various other vital processes, such as intermediary metabolism, Ca^2+^ signaling, and apoptosis. The inevitable production of ROS by mitochondria induces mitochondrial and nuclear DNA damage. Accumulation of damaged DNA and proteins in mitochondria threatens the physiological function of the cell and provokes several defense mechanisms. By fission and fusion processes mitochondrial damage can be partially counteracted and healthy mitochondria might remain functional despite continuous ROS production. Alternatively, damaged mitochondria release so‐called mitochondria‐derived vesicles (MDV), which may be transported to other organelles, such as peroxisomes and lysosomes, in order to restore mitochondrial activity. On the other hand, MDV can be released to the extracellular space as components of the SASP to signal mitochondrial damage to surrounding cells. However, in case of excessive mitochondrial damage, eliminating parts of the organelle is not sufficient and whole mitochondria have to be removed by mitophagy. Dysregulation of mitochondria quality control process leads to accumulation of impaired mitochondria and induction of senescence via various mechanisms.

Reactive oxygen species (ROS) are generated continuously by mitochondria as the result of oxidative metabolism [23] and are known to cause damage to DNA, proteins, and lipid complexes, including mitochondria themselves [21], leading to impaired cellular function and eventually cell death [24, 25, 26, 27]. Besides mitochondria, peroxisomes contribute to cellular ROS levels (Fig. 2). A more detailed description of the interactions between mitochondria and peroxisomes is provided in chapter 3.

Mitochondrial DNA is highly vulnerable to oxidative stress due to its proximity to the electron transport chain (ETC) and due to the limited DNA repair capacity within mitochondria [28]. Perturbation of mitochondrial homeostasis induced by genotoxic stress, ROS and defects in the ETC, among other factors, promotes cellular senescence by activating tumor suppressor pathways like p16^INK4a^/Rb and p53/p21 [29]. Accordingly, senescent cells are characterized by increased mitochondrial ROS production and by metabolic changes related to mitochondrial metabolites and dynamics, mainly attributed to accumulation of dysfunctional mitochondria [30] (Fig. 2).

Senescence caused by mitochondrial dysfunction displays a specific secretory profile, characterized by the absence of factors related to interleukin‐1 (IL‐1), and can be reversed by supplementation with pyruvate [20], suggesting that shortage of oxidized nicotinamide adenosine dinucleotide (NAD^+^) may impair the synthesis of metabolites required for cell proliferation, thereby inducing a specific subtype of senescence. Thus, manipulation of mitochondrial metabolism through the supplementation of intermediate compounds of the respiration process represents a promising target to be explored in order to counteract senescence.

Mitochondrial quality control is a key process for maintenance of a functional mitochondrial network, which in turn is necessary for adaptive metabolism and survival in response to cellular stress (Fig. 2). Besides fission and fusion cycles, a major component of cellular control of mitochondrial integrity is a specialized form of macroautophagy, known as mitophagy, in which mitochondria are specifically targeted for autophagic degradation [31, 32, 33, 34]. A group of mitophagy receptors, including BCL2/adenovirus E1B 19 kDa protein‐interacting protein 3 (BNIP3), BNIP3‐like (BNIP3L/Nix), PTEN Induced Kinase 1 (PINK1), Parkin, and FUN14 Domain Containing 1 (FUNDC1) has been described in recent years. However, more research will be required to delineate redundant and nonredundant functions of known mitophagy receptors. It also seems conceivable that additional members of this family are still to be discovered. Mitophagy plays an important role in cellular homeostasis by eliminating dysfunctional mitochondria and reducing mitochondrial mass as an adaptive response to stress [35]. In mammals, this process is also important during the differentiation of specific cell types such as erythrocyte maturation, mediated by the mitophagy receptor BNIP3L/Nix [36] and for T‐lymphocyte development [37].

A decline of mitophagic activity is related to aging and neurodegenerative diseases, highlighting the pivotal role of mitochondria quality control in maintenance of longevity [38, 39]. In fact, the potential involvement of dysregulated mitophagy in cellular senescence has been suggested by several studies. For instance, some authors have correlated the accumulation of aberrant mitochondria in senescent cells to insufficient turnover of mitochondrial population due to decreased mitophagic activity and lysosomal dysfunction [30, 40]. Likewise, dysfunctional Dnm1l (dynamin 1 like, also known as Drp1)‐dependent mitophagy triggers senescence and contributes to age‐related hearing loss in mice [41]. Yet, in a model of 3D skin equivalents, pyruvate protected dermal fibroblasts from senescence by improving mitophagic activity and mitochondrial turnover [42]. Thus, mitophagy seems to be an important factor for modulation by senolytic and senostatic compounds.

It is important to note, however, that the reasons for the decline in mitophagic activity during the senescence process are not yet fully understood. In some cases, for example, lysosomal dysfunction appears to be related to the accumulation of damaged mitochondria [40]. Others have reported that stabilization of p53 in the cytoplasm during senescence increases its interaction with Parkin and prevents priming of damaged mitochondria, thereby reducing mitophagic activity [43]. Considering that activation and control of mitophagy depends on the specific stress to which cells are subjected during the senescence process [44], it seems to be crucial to understand the underlying mechanisms of mitophagy in different types of senescence and their contribution to the development of the senescence phenotype in order to clarify whether mitophagy dysfunction is a cause or a consequence of senescence.

Notably, parts of mitochondria are also found in so‐called ‘mitochondrial‐derived vesicles’ (MDVs) (Fig. 2). In addition to mitophagy, which is believed to have evolved as a mechanism for degradation of entire mitochondria, MDVs have been proposed as an additional means of quality control for mitochondria [45, 46]. Although the mechanisms involved in the formation and processing of MDVs are still poorly understood, some studies speculate that these vesicles may be part of the SASP and, thus, participate in the processes of interorganelle and intercellular communication during cellular senescence [45] (Fig. 2). Other aspects related to MDVs and senescence are covered in chapter 3. An overview of mitochondria related senescence pathways is given in Table 1.

**Table 1:** Main senescence‐related processes and proteins covered in the review.

Process	Chapter	Proteins/Protein complexes
ROS‐driven pathways	2	p16/pRB, p53/p21
Mitophagy	2	BNIP3, BNIP3L/Nix, PINK/Parkin, FUNDC1
Ca^2+^ signaling/ROS	3	MCU
Mitochondria–lysosome	3	AMPK, RAB7, vCLAMP, Vps13‐Mcp1, Vps39‐ypt7‐Tom40
Mitophagy, autophagy, and general proteostasis	4	Insulin, IGF‐1, DIRAS3, Akt, mTORC1
Ribosome biogenesis and protein synthesis	4	Pol I, mTOR, SirT1, eNoSC, METTL5, 4EBP, elF4G,
Tumour suppressor loss, oncogene activation	5	Sprouty1, Ras, DIRAS3, PI3K, Akt, mTORC1

## Mechanisms of interorganelle communication in cellular senescence

**Fig. 3:**
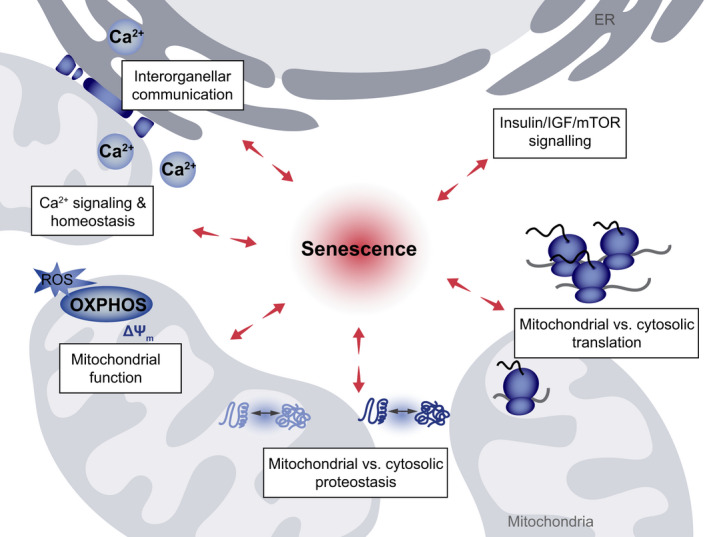
Perturbations of interorganellar connectivity in senescence. Several interconnected processes are affected by cellular senescence, and *vice versa* are impacting the induction and maintenance of the senescent state. These processes include interorganellar communication at ER–mitochondria contact sites and spatially restricted Ca^2+^ signaling occurring at these contacts, as well as general Ca^2+^ homeostasis and transport. The coordination of mitochondrial *versus* cytosolic translation output as well as the interconnected mitochondrial and cytosolic proteostasis networks, all affected by general mitochondrial function and governed by the Insulin‐, Growth Hormone (GH)/Insulin‐like Growth Factor‐1 (IGF‐1)‐, and mechanistic Target of Rapamycin (mTOR)‐signaling axis, act as determinants of senescence induction.

Mitochondria form a highly complex and dynamic network throughout the cytoplasm. They continuously move along microtubule tracks, alter their shape by fusion and fission, and dynamically establish contact sites with other compartments to meet metabolic requirements of the host cell [47, 48]. Apart from changes in the extra‐ and intramitochondrial concentration of signaling molecules, such as Ca^2+^ [49] or ROS [50], the interplay between mitochondria and other organelles occurs through physical proximity at membrane contact sites or via vesicular transport [51] (Fig. 3). For instance, there is evidence for regulatory crosstalk between cytosolic and mitochondrial ribosomes leading to coordinated messenger RNA translation in both mitochondria and the cytosol. Furthermore, the insulin/IGF/mTOR (mammalian target of Rapamycin) axis controls both mitochondrial function and protein synthesis (Fig. 3, for details, see chapter 4). The activity of mitochondria is governed by various mechanisms. Among others, Ca^2+^ ions are necessary for metabolic activation of mitochondria due to the Ca^2+^ dependency of tricarboxylic acid (TCA) cycle dehydrogenases. However, mitochondrial Ca^2+^ overload also triggers cell death pathways. Several studies have revealed changes in the protein machinery controlling mitochondrial Ca^2+^ uptake during aging. For instance, the expression of the mitochondrial Ca^2+^ uniporter (MCU) was increased in long‐term cultured rat hippocampal neurons, leading to elevated mitochondrial Ca^2+^ levels [52]. Moreover, an increase in MCU channel activity was found after oxidation of MCU by ROS [53], commonly elevated during aging [54]. In addition, enhanced ER to mitochondrial Ca^2+^ flux has been shown to boost mitochondrial metabolism in aged endothelial cells, but also bears the risk for mitochondrial Ca^2+^ overload [55].

Consequently, we assume that mitochondrial activity is fine‐tuned by mitochondrial Ca^2+^ homeostasis. Proper mitochondrial Ca^2+^ uptake might be essential to maintain cellular function, while elevated levels of mitochondrial Ca^2+^ might trigger generation of ROS, formation of the permeability transition pore, cytochrome C release [56], and the cell's susceptibility to agents stimulating mitochondrial Ca^2+^ uptake [55]. However, mitochondrial Ca^2+^ overload might be beneficial to overcome the resistance of senescent cells to programmed cell death pathways, which is required for normal cell turnover and tissue homeostasis [57].

Spatially separated cellular subdomains facilitate interorganelle communication. For instance, mitochondria‐associated ER membranes (MAMs) stretch closely to mitochondria to ensure locally restricted and protected ion and lipid transfer between these two organelles in eukaryotic cells [58]. Components of these MAM regions are highly conserved throughout different tissues and species [59]. For instance, a counterpart of these contact sites, so‐called ER‐mitochondria encounter structure (ERMES), was also found in yeast [60, 61, 62]. MAM microdomains are established by reversible tethering of proteins and are equipped with highly specialized toolkits to modulate a variety of cellular processes, including lipid transport and synthesis, Ca^2+^ signaling, autophagy, and energy metabolism [63]. Changes in the ER‐mitochondrial crosstalk have been associated with a reduced adaptive capacity of cells in response to stress and with an increased vulnerability to age‐related diseases [64], including neurodegenerative [65], cardiovascular and metabolic diseases [66], and cancer [67]. Currently, it is a pressing question how substructural composition, dynamics, and function of MAM regions change during the process of aging and which proteins might serve as targets to potentially modulate age‐related cellular dysfunction. We assume that age‐related alterations in the kinetics and dynamics of mitochondrial–ER interaction contribute to the development of cellular dysfunction during senescence. Constant improvement of super‐resolution microscopy techniques may provide the necessary tools to resolve the spatial mitochondrial–ER interplay in real‐time.

Besides ER‐mitochondrial crosstalk, the bidirectional communication between mitochondria and lysosomes has gathered special attention during the last years. As executors and regulators of autophagy, lysosomes are in a crucial position to modulate age‐related pathologies. By preventing the accumulation of damaged mitochondria through mitophagy, lysosomes protect cells from detrimental mitochondria‐derived ROS and pro‐apoptotic factors [68]. Mitochondrial biogenesis was found to be transcriptionally repressed in lysosomal lipid storage diseases, pointing to the immense impact of lysosomal activity on mitochondrial function [69]. In turn, short‐term mitochondrial stress induces lysosomal biogenesis via the regulation of transcription factors and AMP‐activated protein kinase (AMPK) signaling, while chronic mitochondrial stress results in the impairment of lysosomal biogenesis [70]. Moreover, mitochondrial respiratory chain deficiency was found to inhibit lysosomal function via AMPK deactivation and lysosomal Ca^2+^ accumulation [69]. Besides various bidirectional signaling pathways, direct contact sites between mitochondria and lysosomes do exist too. The GTP‐bound lysosomal protein RAB7 was found to promote the formation of these contact sites in healthy cells. Mitochondria tend to undergo fission at lysosomal contact sites, while lysosomal RAB7, in turn, gets regulated by mitochondria [71]. Lysosome–mitochondria communication is also conserved in yeast cells, where contacts between mitochondria and the vacuole (the yeast equivalent of the lysosome) have been described [72], referred to as vCLAMP (vacuole and mitochondria patch). These contact sites can be established by two distinct tethering pairs: Vps13‐Mcp1 or Vps39‐Ypt7‐Tom40 [73].

There is no doubt regarding the crucial impact of mitochondrial–lysosomal interplay on the process of aging as well as on the development of age‐related diseases. For instance, impaired mitochondria–lysosome crosstalk was associated with neurodegenerative diseases [74]. However, it has to be clarified how the composition and dynamics of the lysosomal–mitochondrial contact and interaction sites change during the process of aging and which proteins function as the key regulators of this interplay. In this regard, the utilization of newly developed lysosomally targeted biosensors [75] might represent the key technology that will allow progress in the respective research. Besides ER and lysosomes, latest reports also suggest a direct communication of mitochondria with other cellular sites and organelles, including plasma membrane [76], peroxisomes [77], and endosomes [78].

Mitochondria are frequently found in close proximity to the plasma membrane and contribute to the ATP supply and Ca^2+^ signaling in these specific regions [76]. For instance, mitochondria help to maintain and activate store‐operated Ca^2+^ entry (SOCE), by which depletion of ER Ca^2+^ stores induces Ca^2+^ influx through the plasma membrane [79]. Mitochondria and peroxisomes are both characterized by great plasticity and play a major role in cell metabolism and ROS homeostasis [77]. Notably, direct interorganelle crosstalk between mitochondria and peroxisomes has been reported. For instance, enhanced ROS production in peroxisomes disturbs mitochondrial ROS homeostasis and causes mitochondrial fragmentation. Moreover, peroxisomes and mitochondria share key components of their fission machineries [80].

Recent research connects peroxisomal dysfunction to fatal oxidative damage associated with aging‐related diseases. It is now widely accepted that mitochondria and peroxisomes are required to maintain oxidative balance in a cell. However, our understanding of the interdependence of these organelles to maintain cellular homeostasis of ROS is still limited [81]. A direct interaction between mitochondria and endosomes was shown by super‐resolution microscopy, suggesting alterations in the dynamics of endosome–mitochondrial interaction by endosomal cargo and milieu [78]. However, age‐related alterations in mitochondria's interaction with the plasma membrane, peroxisomes and endosomes are still elusive and need to be characterized.

Extracellular vesicles (EVs) are important mediators of intercellular and potentially interorganellar communication. They transport their functional cargo, which might be composed of proteins, lipids, DNA, and various types of RNA to recipient cells, and thereby act in a similar manner as hormones or cytokines [82]. Recently, small EVs containing miRNAs were identified as important components of the SASP, mediating apoptosis and wound closure [83, 84]. It has been reported that MDVs can transport‐specific mitochondrial proteins and lipids to peroxisomes or lysosomes and act as a first line of defense against mitochondrial damage and preventing complete elimination of the organelle by mitophagy [46, 51]. Hence, mitochondria can be even found in the extracellular environment in their free form, or surrounded by a membrane (like in vesicles) [45, 51, 85]. Moreover, circulating cell‐free mitochondrial DNA has been found as well [46]. Notably, all forms of extracellular mitochondria have been found to induce paracrine or endocrine responses in various organisms. However, the effects of extracellular mitochondria just start to be elucidated [46, 51] and their impact on processes of senescence is still elusive. Now, latest developments in super‐resolution microscopy are allowing us to catch a glimpse of mitochondria's versatile interaction with their cellular environment and we might be curious what will be unveiled in the following years. An overview of pathways related to disturbed interorganelle communication during senescence is given in Table 1.

## Mechanisms of metabolic control and proteostasis in cellular senescence

The energy status of a cell and the availability of nutrients represent the most important upstream regulators of mitochondrial dynamics and quality control. These, as well as other processes of metabolism and accumulation of biomass, are assured by the mammalian target of rapamycin (mTOR) pathway at the nexus of nutrition, cell growth, and aging [86, 87]. Metabolic interventions known to extend healthy life span, such as caloric/dietary restriction, time‐restricted feeding, and intermittent fasting all affect the main nutrient sensing pathways including mTOR. Therefore, mTOR holds a central position to integrate aging‐associated processes. Over‐activation of mTOR leads to induction of cellular senescence, while repression of mTORC1 by rapamycin or genetic interventions blocks cellular senescence [88, 89], attenuates the SASP [90], and extends the life span of mice [91, 92, 93]. Besides mitophagy [94], mTORC1 also regulates general autophagy, as well as other processes of both the anabolic and catabolic arm of cellular proteostasis. Of interest, previous work from the Jansen‐Dürr laboratory established a tight link between mitochondrial dysfunction and functionality of the ubiquitin‐proteasome system. Thereby, a major mechanism of catabolic proteostasis was found associated with human skin aging, providing a mechanistic link between mitochondrial quality control and proteostasis [95].

**Fig. 4:**
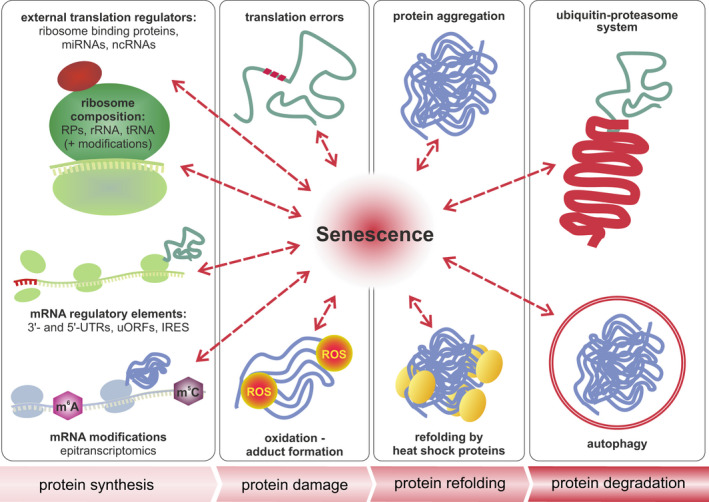
The role of senescence in multilevel proteostasis—the four stages in the life of a protein. Cellular senescence and aging presumably influence all four stages in the life of a protein, namely its synthesis by ribosomes, the accumulation of damage, refolding, and aggregation, as well as its degradation. Protein synthesis is tightly controlled by the composition of ribosomes, association of the core translation machinery with external regulators, mRNA modifications, and specific sequence elements of mRNAs, such as the 3′ and 5′ untranslated regions (UTRs), short upstream open reading frames (uORFs), or internal ribosome entry sites (IRES). Translation errors and oxidation lead to damaged and misfolded proteins, which are prone to aggregation. This process is counteracted by heat shock proteins or molecular chaperones. Damaged and aggregated proteins are degraded by autophagy or via the ubiquitin‐proteasome system. Both refolding and degradation are impaired in cellular and organismal aging and are tightly interconnected with other stages of proteostasis.

Cellular proteostasis is guaranteed by the balance between the synthesis of new proteins, the impact of protein damaging mechanisms versus cellular defence mechanisms, protein refolding by chaperones and related pathways, and the clearance of damaged proteins (Fig. 4). Thus, timely modulation in response to extrinsic stimuli, and correct function of all four interconnected stages of protein turnover and quality control are essential for organismal health.

Accumulating evidence suggests all levels of proteostasis to be altered when cells enter senescence. The importance of protein misfolding, aggregation, and subsequent clearance by the ubiquitin‐proteasome system or autophagy for healthy aging and senescence‐related pathologies is widely recognized and extensively reviewed elsewhere [96, 97, 98]. Although depletion of ribosomal proteins and other factors involved in translation are known to extend the life span of yeast [99, 100, 101] and *C. elegans* [102, 103, 104], their potential role in mammalian cell senescence and the underlying molecular mechanisms are by far less understood than other aspects of cellular proteostasis [105].

Current hypotheses postulate that decreased overall translation conserves energy, which can then be invested into maintenance and repair pathways [106] or that reduced elongation speed minimizes translation errors [107]. Both mechanisms, by increasing the overall protein repair capacity, may limit the age‐related accumulation of oxidized, misfolded and aggregated proteins. At the same time, reduced amounts of mis‐translated polypeptides do not unnecessarily block important refolding and degradation pathways [108].

Due to the high energy demands of protein synthesis, it is not surprising that also ribosome biogenesis is tightly controlled by nutrient sensing pathways. Again, mTOR plays a pivotal role by repressing pre‐rRNA transcription by RNA Polymerase I (Pol I) when nutrients are limited. SirT1 is another conserved nutrient sensor linking aging to Pol I activity. Sir2, the yeast orthologue of SirT1, was originally identified as one of the first genes modulating cellular aging by maintaining rDNA integrity [109]. Although the finding that excision of rDNA repeats and the formation of extrachromosomal rDNA circles limit replicative life span is restricted to yeast, a large body of evidence indicates that rRNA transcription by Pol I is also a critical factor in aging of higher eucaryotes [110]. Partial inhibition of Pol I extends life span of the fruit fly and leads to shrinkage of nucleoli, which are clustered around rDNA repeats and represent the sites of ribosome biogenesis [111]. Also, in senescent human cells several small nucleoli fuse to one large nucleolus, indicating profound changes in the chromatin organization of rDNA repeats. Their silencing by the SirT1‐containing eNoSC‐complex and inhibition of Pol I is required for maintaining the senescent state of A549 cancer cells [112]. Interestingly, rDNA integrity is also tightly linked to several human segmental progeroid syndromes. Mutations in distinct RecQ helicases inflict the respective pathologies characterized by high levels of rDNA recombination and rearrangements [110]. Syndrome, for instance, is caused by recessive mutations in five different nucleotide excision repair proteins. Mutations in all five of these proteins cause defects in Pol I‐mediated transcription and translational fidelity, leading to elevated oxidative protein damage [113]. Taken together, these data indicate a striking connection between proteostasis, nutrient sensing, DNA integrity, and mitochondria, which all represent different hallmarks of aging [114].

In addition to the outlined changes in their biogenesis, it is generally believed that ribosomes and other components of the translation machinery deteriorate with age, leading to decreased overall protein synthesis and progressive loss of gene expression regulation at the translation level. Indeed, an increasing number of studies report certain discrepancies between the transcriptome and the proteome, which indicates that profound changes of translational regulation of gene expression occur during aging [115].

We hypothesize that selection of different mRNAs for translation occurs due to alterations in stoichiometry and modification patterns of several components of the translation machinery, which might all be influenced by aging and cellular senescence (Fig. 4). The essential components include the ribosome itself, as well as mRNAs, tRNAs, initiation, and elongation factors. In addition, multiple ribosome‐associated proteins, miRNAs and ncRNAs, such as tRNA fragments and rancRNAs, are described, which can modulate translation by competing with essential components or by allosteric interactions [105]. Indeed, known interventions modulating aging, stress resistance, and cellular senescence alter the translational regulation of gene expression. In flies, it was, for example, shown that the life span extension by deletion of Thor (also known as 4E‐BP) is modulated by the specific translation of mRNAs associated with enhanced mitochondrial activity [116]. Similarly, in worms the translational regulation of stress response genes is required for the life span extension by deletion of eukaryotic translation initiation factor 4G (eIF4G) [117]. In contrast to these protein‐based alterations in ribosome composition and functionality, also lack of a single, conserved N5‐cytosine methylation of ribosomal RNA of the large subunit extends the life span and stress resistance of yeast, worms, and flies. Depletion of the corresponding methyltransferase NSUN5 alters ribosomal structure and thus translational fidelity, resulting in a ‘reprogramming’ of the ribosome towards translation of mRNAs involved in cellular stress response [118]. Similarly, N6‐adenosine methylation of a specific residue of 18S rRNA by methyltransferase like 5 (METTL‐5) modulates heat‐stress resistance in *C. elegans* by altering translation of a specific mRNA involved in eicosanoid synthesis [119]. With the proof‐of‐principle that already single modifications of rRNA alter the life span and stress tolerance of organisms, it becomes clear that a systematic analysis of global post‐transcriptional modification patterns of rRNA, including pseudouridinylations, as well as base and sugar methylations [120], is of crucial importance to understand the changes of the ribosome in terms of synthesis, composition, structure, and function in the context of aging. In a similar fashion to rRNA modifications, also mRNA modifications influence translation [121] and it is tempting to speculate that these might be regulated in cellular and organismal aging as well.

An additional layer of complexity is added by mitochondrial ribosomes contained in eukaryotic cells in addition to the ribosomes in the cytosol. Both types of ribosomes appear to be coordinated, and this balance between cytosolic and mitochondrial translation may be altered under stress, and in senescent cells in particular [122, 123, 124, 125]. Methionine restriction, which increases the replicative life span of human fibroblasts, differentially affects the translation of selected mitochondrial RNAs [126] and thereby demonstrates the complex interplay between metabolic regulation, proteostasis, and mitochondria. Thus, we hypothesize that certain metabolic interventions might selectively target senescent cells by altering coordinated multilevel proteostasis of both cellular compartments. Moreover, a thorough analysis of age‐related alterations of the protein synthesis machinery might provide us with promising novel targets for anti‐senescence therapies. An overview of senescence pathways related to disturbed proteostasis and metabolic regulation is given in Table 1.

## Identification of dietary and pharmacological interventions modulating cellular senescence

Restoring normal cellular and tissue function in an aged organism can be achieved by several strategies, including counteracting senescence‐inducing signals, targeting of specific aging‐related mechanisms, eliminating senescent cells (senolysis), and suppressing or switching senescence‐associated phenotypes including the SASP by senostatics/senomorphics [127, 128, 129].

### Simple eukaryotic model organisms as potential tools for senescence effector screening

Simple eukaryotic model organisms, including *S. cerevisiae* and *C. elegans*, have been used as conventional tools for genetic screens concerning senescence effectors [130, 131, 132]. In *S. cerevisiae*, critical telomere shortening upon genetic inactivation of the telomerase, for example, via depletion of one of its subunits (Est1, Est2, Est3, or Tlc1), leads to an abrupt transit into replicative senescence after about 50–80 cell divisions (recently reviewed by [132, 133]). Like in mammalian cells, this is accompanied by a permanent activation of the DNA damage checkpoint, reorganization of chromatin, and prominent changes in gene expression [11, 133, 134]. Interestingly, genes related to mitochondrial energy metabolism, ranging from oxidative phosphorylation to TCA cycle, have been found to be prominently upregulated in senescent yeast [135]. In addition, mitochondrial proliferation is increased, indicating that also in this unicellular eukaryote, mitochondrial metabolism and functionality may contribute to the induction and/or maintenance of the senescent state [135]. Although both yeast and *C. elegans* lack complex traits associated with senescence, these models contributed substantially to our current understanding of fundamental molecular aspects associated with aging and senescence [11, 12, 15, 132]. Genes identified in such screens were subsequently validated in higher eukaryotic model systems, such as human cultured cells [55, 136, 137], organoids [138, 139], and mice [140, 141], and promoted the identification of promising molecules for translational approaches [55]. It is conceivable that many more senescence effectors from the chemical space can be identified by exploiting existing and newly defined senescence regulators as target proteins in small‐molecule screens.

### CR mimetics as antisenescence drugs

The employment of caloric restriction (CR)/dietary restriction (DR) mimetics [142] may represent one promising strategy for pharmacological targeting of senescent cells. The most robust procedure to counteract aging is CR, the reduction of dietary intake below energy requirements while maintaining adequate nutrition [143, 144]. Although CR is a complex intervention with many experimental variables, including sex, strain, and level of CR, that can alter the CR response in model organisms, it consistently improves health across strains and sexes [145]. Molecular mechanisms underlying CR are not completely understood. According to the current model, the CR response is transduced via modulation of nutrient‐ and energy‐signaling pathways mainly inducing a reduction of Insulin/growth hormone (GH)/insulin‐like growth factor 1 (IGF‐1)/mTOR signaling and an activation of AMPK‐signaling and Sirtuin responses [146, 147]. This drives many downstream changes such as reduced oxidative stress and increasing stress resistance leading to less damage of DNA [148, 149, 150], proteins [151], and lipids [152] (for reviews, see [153, 154]). Most likely these events contribute to the prevention of age‐associated decline in genomic stability, autophagy, and proteostasis. Additional beneficial effects are that CR preserves mitochondrial function with age by increasing mitochondrial biogenesis, reprogramming of metabolism to anaplerotic filling of the TCA cycle and activation of fatty acid oxidation in mitochondria [155]. While there is consensus that CR increases health span in a wide variety of animal models, evidence from observational and randomized controlled clinical studies conducted in the last two decades suggests that CR can not only extend the healthy life span of obese and overweight people [156] but also of normal weight persons and might have the potential to induce longevity in humans under CR [157, 158].

**Fig. 5:**
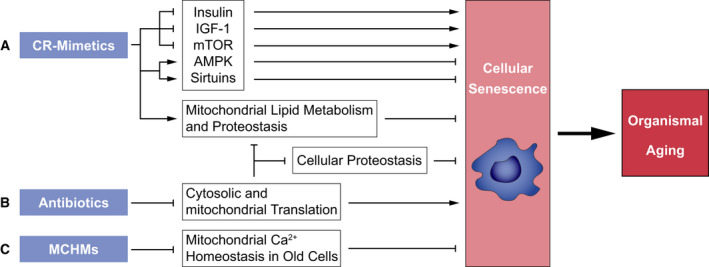
Potential dietary and pharmacological compounds modulating cellular senescence. Mitigating the induction and propagation of cellular senescence by (A) caloric restriction (CR)‐mimetics and (B) antibiotics targeting cytoplasmic and mitochondrial translation. Both compound classes counteract senescence‐inducing signals. (C) Mitochondrial Ca2+ homeostasis manipulators (MCHMs) could act senolytic. AMP‐activated protein kinase (AMPK), ER = Endoplasmic Reticulum, Insulin‐like growth factor‐1 (IGF‐1), mechanistic target of rapamycin (mTOR), Sirtuins (NAD+‐dependent deacetylases).

Given the importance of CR for human health, translational research into screening for and developing of CR mimetics, compounds that mimic the positive effects of CR on health and life span without actual food restriction [159], is an appealing strategy. While CR works most likely on several different levels [160], the question whether and how CR antagonizes cellular senescence is one current focus of aging research [161]. Evidence supporting the hypothesis that CR mitigates or reduces cellular senescence comes from studies showing that under CR senescence markers are reduced in mouse and human organs [89, 170]. As it is well known that CR protects against cellular damage induced by oxidative stress and other adverse influences [147, 155], and cellular damage is a major cause for the induction of cellular senescence [171], it is quite likely that CR counteracts the generation of senescent cells by reducing cellular damage. It has been demonstrated that CR ameliorates senescence‐associated DNA damage and induces a decrease of the number of senescent cells in several tissues of mice [162, 163, 164, 169, 172]. The Zwerschke‐lab has shown that weight‐loss (WL) interventions including CR protects human adipose stem/progenitor cells (ASCs) against DNA damage and prolongs their life span by postponing the onset of cellular senescence [164]. Moreover, they identified WL/CR target genes, DIRAS family GTPase 3 (DIRAS3) and Sprouty1, that protect against the induction of cellular senescence [89, 166, 167, 170]. Mechanistically, DIRAS3 and Sprouty1 act by reducing signaling from the two main aging inducing signal transduction pathways, insulin/phosphoinositide 3 kinase (PI3K)/Akt/mTOR signaling, and IGF‐1/ras/mitogen‐activated protein kinase (MAPK) signaling, leading to protection against cellular senescence. Moreover, the activity of the PI3K inhibitor DIRAS3 leads to downregulation of mTOR activity and in turn to the stimulation of autophagy, a process that is well known to recycle damaged cellular components [170]. Thus, one could refer to the CR target gene DIRAS3 as a recycling and/or a cellular rejuvenation gene postponing cellular senescence (Fig. 5). Given that the induction of DIRAS3 and Sprouty1 in response to CR is sufficient to turn off signaling from the central Insulin/IGF‐1/mTOR pro‐aging program, one could speculate that at least some cell types in given tissues or organs are protected by cell intrinsic antisenescence mechanisms, which might act in combination with or independent of Insulin and IGF‐1. It has also been shown that CR in humans protects against cellular deterioration via decreasing oxidative stress and eliminating present damage by increasing the expression of genes encoding for heat shock proteins and proteins involved in autophagy [173, 174]. Moreover, it was previously shown that CR inhibits mTOR activity and hence abrogates the mTOR‐dependent pro‐inflammatory phenotype of senescent cells including factors that induce bystander senescence [90, 175], underscoring that CR could prevent the activation of primary and secondary senescence by inhibiting mTOR.

It is well accepted that genetic or pharmacological inhibition of mTORC1 by rapamycin or rapamycin‐derived compounds (rapalogs), postpones aging and increases the life span of a wide variety of animals including mice [91, 176, 177, 178, 179, 180, 181]; and although there may be exceptions [182], our understanding of the current literature is that rapamycin and the majority of related compounds (rapalogs) are actually dampening the senescence response. Additional mechanisms by which CR can prevent cellular senescence are the elimination of dysfunctional mitochondria by mitophagy [183] and activation of DNA repair mechanisms [184, 185].

Thus, there is overwhelming evidence supporting the hypothesis that CR mimetics can counteract cellular senescence. Our recent studies showing that WL interventions, including CR, postpone the onset of replicative senescence of ASCs in humans [164, 186] and identifying CR target genes in these cells that are involved in the regulation of cellular senescence pathways [89, 166, 167, 170] provided novel candidate molecules for the development of CR mimetics. We hypothesize that CR mimetics identified in such an approach are also active in modulating cellular senescence. Such compounds should have the potential to counteract senescence‐inducing signals and to suppress and/or modulate senescence‐associated signaling, thereby protecting cells and tissues in our body and maintaining physiological functions longer.

### Calcium signaling and mitochondria as targets for antisenescence interventions

Another promising strategy to target senescent cells is the manipulation of mitochondrial Ca^2+^ homeostasis. The tight linkage between mitochondria and the ER strongly impacts the susceptibility of aged endothelial cells to the cytotoxic effects of agents inducing mitochondrial Ca^2+^ overload, such as polyphenols [55]. Studying this mechanism might lead to a potentially new class of senolytics based on mitochondrial Ca^2+^ overload. In a similar way, compounds previously selected for regulating mitochondrial activity in various senescence models have the potential to modulate mitochondrial physiology in order to overcome/target specific types of senescence, such as mitochondrial dysfunction‐associated senescence. In such settings, pharmacological modulation of mitochondrial fitness can be evaluated by analysis of mitochondrial fragmentation, mitophagy, and ROS production. Both approaches bear the promise to develop novel senolytic and or senostatic drugs (Fig. 5).

### Proteostasis optimizers for antisenescence interventions

Dampening of protein synthesis may represent another promising strategy for pharmacological targeting of senescent cells. As many antibiotics targeting eukaryotic cells selectively inhibit different steps of cytosolic or mitochondrial translation [187, 188], we hypothesize that at least some of these compounds may postpone senescence and mitigate adverse effects of the SASP by reducing protein synthesis (Fig. 5). However, whether promising drug candidates act via repression of global protein synthesis, via promoting the selective translation of specific anti‐senescence mRNAs, or via so far unknown mechanisms, will also remain to be elucidated.

## Conclusions and perspectives

The discovery in genetically modified mice that senescent cells drive aging in animal models [189] has spurred huge research attempts to find pharmacological tools that may promote healthy aging by elimination of senescent cells (‘senolysis’) or disabling/switching their function in human tissues (‘senostasis’, ‘senomorphism’). In parallel, enormous research efforts with the goal to understand the biology of cellular senescence have highlighted relevant molecular pathways of senescence. These discoveries may lead the way to unveil potential pharmacological targets. Maintaining cells in various human tissues healthy and fully functional seems to require a sophisticated interplay between various cellular organelles, highly efficient quality control of mitochondria, functional interorganelle communication, and a tight regulation of proteostasis at various levels. This fragile system is challenged by internal and environmental stress factors causing failures of single components of this network that may be sufficient to drive a cell into a pathological state known as cellular senescence. A better understanding of the hierarchy of and synergy between the various signaling pathways of senescence bears the promise to identify new critical targets for antisenescence interventions.

## Conflict of interest

The authors declare no conflict of interest.

## Author contributions

MC, CTM‐S, SB, MS, WZ, SW, WFG, JG, and PJ‐D collectively wrote the review and read, as well as edited, the entire manuscript.
